# Burden of hereditary cancer susceptibility in unselected patients with pancreatic ductal adenocarcinoma referred for germline screening

**DOI:** 10.1002/cam4.2973

**Published:** 2020-04-07

**Authors:** Carol Cremin, Michael Kuan‐Ching Lee, Quan Hong, Carolyn Hoeschen, Anna Mackenzie, Katherine Dixon, Mary McCullum, Jennifer Nuk, Steve Kalloger, Joanna Karasinska, Charles Scudamore, Peter T. W. Kim, Fergal Donnellan, Eric C. S. Lam, Howard J. Lim, Cynthia L. Neben, Will Stedden, Alicia Y. Zhou, David F. Schaeffer, Sophie Sun, Daniel J. Renouf, Kasmintan A. Schrader

**Affiliations:** ^1^ Hereditary Cancer Program BC Cancer, part of Provincial Health Services Authority Vancouver BC Canada; ^2^ Pancreas Centre BC Vancouver BC Canada; ^3^ Division of Medical Oncology BC Cancer, part of Provincial Health Services Authority Vancouver BC Canada; ^4^ Department of Medical Genetics University of British Columbia Vancouver BC Canada; ^5^ Department of Pathology & Laboratory Medicine Vancouver General Hospital Vancouver BC Canada; ^6^ Department of Surgery Vancouver General Hospital Vancouver BC Canada; ^7^ Department of Gastroenterology Vancouver General Hospital Vancouver BC Canada; ^8^ Department of Gastroenterology St. Paul's Hospital Vancouver BC Canada; ^9^ Color Genomics Burlingame CA USA; ^10^ Department of Molecular Oncology, BC Cancer part of Provincial Health Services Authority Vancouver BC Canada

**Keywords:** genetic consultation, hereditary cancer, pancreatic ductal adenocarcinoma

## Abstract

**Background:**

Recent guidelines recommend consideration of germline testing for all newly diagnosed pancreatic ductal adenocarcinoma (PDAC). The primary aim of this study was to determine the burden of hereditary cancer susceptibility in PDAC. A secondary aim was to compare genetic testing uptake rates across different modes of genetic counselling.

**Patients and Methods:**

All patients diagnosed with PDAC in the province of British Columbia, Canada referred to a population‐based hereditary cancer program were eligible for multi‐gene panel testing, irrespective of cancer family history. Any healthcare provider or patients themselves could refer.

**Results:**

A total of 305 patients with PDAC were referred between July 2016 and January 2019. Two hundred thirty‐five patients attended a consultation and 177 completed index germline genetic testing. 25/177 (14.1%) of unrelated patients had a pathogenic variant (PV); 19/25 PV were in known PDAC susceptibility genes with cancer screening or risk‐reduction implications. PDAC was significantly associated with PV in *ATM* (OR, 7.73; 95% CI, 3.10 to 19.33, *P* = 6.14E‐05) when comparing age and gender and ethnicity‐matched controls tested on the same platform. The overall uptake rate for index testing was 59.2% and was significantly higher with 1‐on‐1 consultations and group consultations compared to telehealth consultations (88.9% vs 82.9% vs 61.8%, *P* < .001).

**Conclusion:**

In a prospective clinic‐based cohort of patients with PDAC referred for testing irrespective of family history, germline PV were detected in 14.1%. PV in *ATM* accounted for half of all PVs and were significantly associated with PDAC. These findings support recent guidelines and will guide future service planning in this population.

## Graphical abstract

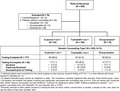

## 1 BACKGROUND

Reported prevalence of likely pathogenic or pathogenic germline variants (PV) in pancreatic ductal adenocarcinoma (PDAC) unselected for family history, has been as low as 3.5% using 13‐ and 32‐gene multiplexed panels, while enrichment of cohorts with cancer family history or Ashkenazi Jewish ancestry can increase rates up to 10.4%‐30%.^1^, ^2^, ^3^, ^4^, ^5^, ^6^ Germline PV are increasingly recognized to have therapeutic implications for the patients themselves (ie, *BRCA1*/*BRCA2* and poly ADP ribose polymerase (PARP) inhibitors) as well as cancer risk‐reduction implications for healthy relatives who can follow established syndrome‐specific management guidelines.^7^, ^8^, ^9^

Patients with PDAC PV are missed with traditional criteria‐based testing due to lack of notable personal or family history or predictive clinicopathological features.^6^ In light of this, the National Comprehensive Cancer Network (NCCN) now recommends consideration of germline testing for all newly diagnosed PDAC.^9^, ^10^ The American Society of Clinical Oncology (ASCO) also issued a provisional clinical opinion in support of this guidance and advocated for research into the utility of multigene panel testing.^11^

At Hereditary Cancer Program (HCP), since June of 2016, we have been conducting clinical grade panel‐based germline genetic testing in unselected pancreatic ductal adenocarcinoma patients referred to a population‐based hereditary cancer program in a publicly funded health care system, which services the 4.6 million‐person population of British Columbia, Canada.

The primary aim of this study was to report on the testing uptake rate and mutation detection rate based on the 305 patients referred over the 2.5‐year study period. A secondary aim was to compare genetic testing uptake rates across different modes of genetic counselling to assist in sustainable delivery and to guide future service planning for unselected testing in this population.

## 2 METHODS

### 2.1 Eligibility

All patients with PDAC diagnosis between July 2016 and January 2019 in the province of British Columbia (BC), Canada, and referred to the HCP, were eligible to undergo clinical‐grade, NGS panel testing, based on their personal history of PDAC alone, irrespective of family history. Diagnoses were confirmed histologically or by clinical and radiologic findings where biopsy was not possible. Any healthcare provider or patients themselves could refer. Patients with PDAC diagnosed prior to July 2016 who had been on the HCP waitlist were also invited to participate and were seen prospectively. Patients referred for carrier testing or confirmatory testing of research findings were excluded from the index cohort analysis.

Genetic counselling appointments (in‐person, by telehealth, or by group session) were offered within 3 months (1 month if urgent) based on patient preference, health status, and geographic location and were seen “1‐on‐1” unless specified as “group.” Patient‐reported outcome measures included a 5‐point Likert scale survey to assess satisfaction that was administered following the group session. This study was conducted under the approval of the BC Cancer Research Ethics Board. Patients attending their appointment in person signed clinical and research consent forms on site and provided their sample using the saliva kit. Patients attending their appointment by telehealth received the clinical and research consent forms by mail after their appointment, along with the saliva kit. These patients were instructed to mail back all consent forms and to arrange for a courier pick‐up of their saliva kit.

### 2.2 Genetic testing

All referred PDAC patients, regardless of family history of cancer, were eligible for a research funded external clinical‐grade, 30 gene saliva‐based NGS panel test for hereditary cancer which included the following genes: *APC, ATM, BAP1, BARD1, BMPR1A, BRCA1, BRCA2, BRIP1, CDH1, CDK4, CDKN2A (p14ARF* and *p16INK4a), CHEK2, EPCAM, GREM1, MITF, MLH1, MSH2, MSH6, MUTYH, NBN, PALB2, PMS2, POLD1, POLE, PTEN, RAD51C, RAD51D, SMAD4, STK11,* and *TP53*.^12^ A subset of patients had a different panel because they met local criteria for publicly funded testing either for *BRCA1*/*BRCA2* testing using an in‐house 17 gene panel or a larger panel with 42‐83 genes if their personal and family cancer history was suggestive of other genetic syndromes. After protocol amendment in July 2018, those patients meeting publicly funded *BRCA1*/*BRCA2* criteria were offered the 30 gene Hereditary Cancer Test. The panels used for each index case are described in Table S1.

### 2.3 Clinical data

A three‐generation pedigree for all patients was obtained to determine whether or not the family history met familial pancreatic cancer (FPC) criteria or NCCN criteria for hereditary testing. FPC criteria were met if a patient reported having a family history in which two relatives had pancreatic cancer and were in a first‐degree relationship to one another (one of the relatives could be the patient themselves). NCCN criteria were met if family history met NCCN *BRCA1/BRCA2* criteria version 2.2017.^13^ Treatment and overall survival outcomes were retrospectively collected through electronic chart review. Diagnosis of diabetes was determined by consultation documentation and/or fasting glucose > 7 mmol/L or HbA1c > 48 mmol/L.^14^ Peripancreatic diabetes was defined as diabetes diagnosed within 3 years prior to PDAC diagnosis. Surgical determination of resectable status (resectable, borderline, locally advanced, metastatic) was determined as per consensus‐based guidelines from the NCCN.^10^ Rates of carrier testing for family members unaffected by PDAC were also assessed.

### 2.4 Case‐control analysis

To control for population substructure impacting PV detection rates, patients who had the Color Genomics Hereditary Cancer Test (N* = *164) were compared to an age (20‐year categories), gender, and ethnicity (European, Asian, Ashkenazi Jewish, and other) matched control cohort from Color Genomics. The control cohort were Color Genomics clients who took the Hereditary Cancer Test, had no personal history of (any) cancer and were not patients of the BC Cancer Agency. Variant frequency from Color Genomics Data can be found at data.color.com/v1.

The ratio of matched controls to our cohort was 9:1, with the exception of “Male, 50‐69, Asian”; “Male, 70‐89, Asian”; and “Female, 70‐89, Asian” subgroups due to inadequate subgroup representation in the control cohort. Therefore, a lower ratio match was obtained for these subgroups. These controls had the Hereditary Cancer Test ordered by a healthcare provider and provided informed consent to have their de‐identified information and sample used in anonymized studies.

### 2.5 Statistical methods

Pearson chi‐squared test or Fisher's exact test was used to compare the differences in the categorical baseline characteristics and a Mann‐Whitney U test was applied to compare the distribution of age at diagnosis between patients with and without PV. To compare the uptake of index testing between counselling types, post‐hoc testing was conducted after the overall Pearson chi‐squared test. Patient survival was analyzed by Kaplan‐Meier method with time to death defined by the time from initial PDAC diagnosis to the time of death. Data for survival was censored on 31 March 2019. For the case‐control analysis, associations between PDAC and each gene were analyzed using Fisher's exact test. Odds ratios (ORs) and 95% confidence intervals (CIs) were calculated. *P*‐values < .05 were considered statistically significant. For post‐hoc testing, statistical significance was set at Bonferroni corrected *P*‐value < .0083. Statistical analyses were performed using SPSS software version 25.0.

## 3 RESULTS

A total of 305 PDAC patients were referred to the HCP between July 2016 and January 2019. Referrals were from oncologists (70.8%, 216/305), surgeons (8.2%, 25/305), general practitioners (7.9%, 24/305), other (6.6%, 20/305), and patient self‐referral (6.2%, 19/305). Six PDAC patients had a known familial PV (5) or research‐identified PV (1) prior to referral and were excluded from the index cohort and genetic testing uptake rates analysis.

**Figure 1:**
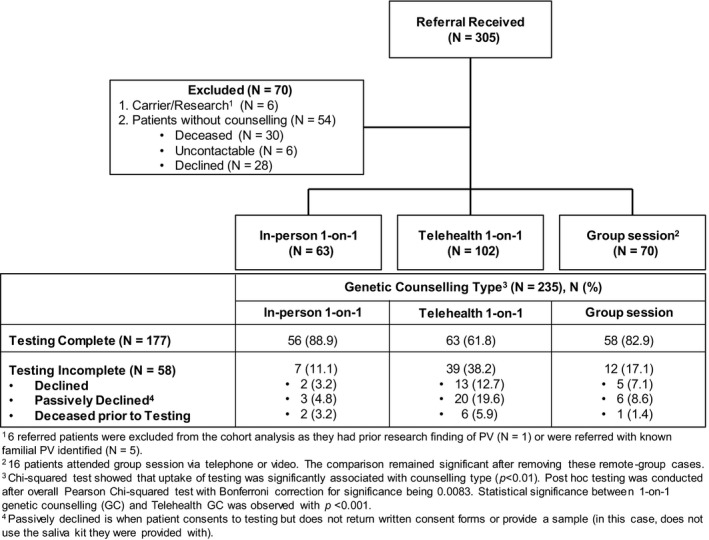
Patient flow

As shown in Figure 1, 77% (N = 235) of PDAC patients accepted a formal genetics consultation either in the form of in‐person 1‐on‐1 consultation (N = 63), telephone/video 1‐on‐1 consultation (N = 102), or group information session (N = 70). Patients were deemed eligible for the group session if they spoke English and lived within an approximated one hour driving distance to the clinic. Patients who declined the group session were offered a 1‐on‐1 consultation in person/video/telephone depending on patient preference and location. Patients attending the group session were provided with direct contact information if they wished to speak 1‐on‐1 with a genetic counsellor regarding personal issues or questions after the session.

Patient satisfaction was measured with a 14 question survey that was previously developed internally in our clinic and assessed for face validity.^15^ The survey was completed anonymously and voluntarily after each group session from 27 October 2016 to the study end period (Table S5). Among 54 completed surveys returned, 53 respondents (97%) stated they agreed or strongly agreed with the Statement 1, “Overall, this appointment was helpful to me.” To compare satisfaction among patients undergoing 1‐on‐1 appointments (in person or by tele/video health), the same survey was administered to all PDAC patients who attended a 1‐on‐1 appointment from 31 January 2019 to 1 July 2019. Thirty‐one surveys were returned and 30 completed the satisfaction question with 30/30 (100%) stating they agreed or strongly agreed with Statement 1.

Overall, 12.8% (N = 39) PDAC patients died before genetic testing could be offered or performed, and 27.2% (N = 83) either declined or passively declined a hereditary cancer assessment (ie, did not accept the genetics consultation or did not consent to testing or did not provide a sample). Of those who accepted genetic consultation, 75.3% (N = 177/235) completed index genetic testing. Overall, 59.2% (N = 177/299) of all referred index patients completed genetic testing. The uptake rate for index testing did not differ between referral sources. Also shown in Figure 1 is a comparison of uptake rate for index testing among different appointment types. The uptake rate with 1‐on‐1 consultations was slightly higher than for group sessions (88.9% vs 82.9%) and telehealth resulted in a significantly lower uptake (61.8%, *P* < .001).

The majority of germline testing (92.6%) was performed with the 30 gene Color Genomics Hereditary Cancer Test. The majority of germline testing (85.3%) was funded under the research protocol. Of the 26/177 cases that met specific criteria and protocols for publicly funded genetic testing, all had the Color Genomics panel except nine that had broader panels and four cases that had more restrictive panels (Table S1). Of the latter four patients, one had a *MSH2* PV detected and further wider panel testing was not offered and the remaining two patients were deceased prior to the protocol amendment therefore wider panel testing was not offered. One patient only had site specific mutation testing to confirm a *BRCA2* mutation detected through another research program that utilized whole genome sequencing. This patient was included in the study as the referral pre‐dated the research finding. For this case, the reason for referral was incident pancreatic cancer and no other high risk features were indicated on referral.

Clinical and demographic characteristics of 177 patients who completed index germline testing are listed in Table 1. Baseline characteristics were similar between the PV and uninformative group (Table 2). The median overall survival of resectable, borderline, locally advanced, and metastatic subgroups were 53 months (95% CI, 26 to 80), 26 months (95% CI, 14 to 38), 16 months (95% CI, 13 to 19), and 13 months (95% CI, 11 to 15), respectively, and there was no statistical difference in survival between the PV and uninformative group (Figure S1).

**Table 1:** Baseline characteristics (N = 177)

Characteristics	All patients N (%)
Age at diagnosis, years (Median, range)	64 (36‐89)
Gender	
Female	98 (55.4)
Male	79 (44.6)
Ethnicity	
European	124 (70.1)
Asian	38 (21.5)
Ashkenzai Jewish	3 (1.7)
Other	7 (4.0)
(Missing data)	5 (2.8)
Diabetes	
Long term (>3 y)	24 (13.6)
Peripancreatic (<3 y)	17 (9.6)
No	136 (76.8)
Prior smoking history	74 (41.8)
Personal history of other cancer	50 (28.2)
Breast cancer	20 (11.3)
Patients with germline PV identified	25 (14.1)
Known susceptibility genes	19 (10.7)
Met FPC^1^ criteria	30 (16.9)
Met NCCN^2^ criteria for BRCA testing	68 (38.4)
Met either FPC or NCCN criteria	82 (46.2)
Stage of cancer	
Resectable	52 (29.4)
Borderline resectable	16 (9.0)
Locally advanced	44 (24.9)
Metastatic	65 (36.7)
Primary resection	60 (33.9)
ECOG status	
0	40 (22.6)
1	91 (51.4)
2	35 (19.8)
3	11 (6.2)

^1^ FPC = familial pancreatic cancer.

^2^ NCCN = National Comprehensive Cancer Network version 2017.

**Table 2:** Comparisons between patients with (N = 25) and without (N = 152) PV identified

Characteristics	PV identified N (%)	Uninformative and VUS N (%)	*P* value
Age at diagnosis, years (Median, range)	64 (36‐ 87)	60 (37‐89)	.493
Gender			.715
Female	13 (52.0)	85 (55.9)	
Male	12 (48.0)	67 (44.1)	
Ethnicity			.810
European	18 (72.0)	106 (69.7)	
Asian	6 (24.0)	32 (21.1)	
Ashkenzai Jewish	0 (0.0)	3 (2.0)	
Other	0 (0.0)	7 (4.6)	
(Missing data)	1 (4.0)	4 (2.6)	
Diabetes			.199
Long term (>3 y)	1 (4.0)	23 (15.1)	
Peripancreatic (<3 y)	1 (4.0)	16 (10.5)	
No	23 (92.0)	113 (74.3)	
Prior smoking history	11 (44.0)	63 (41.4)	.810
Personal history of other cancer	6 (24.0)	44 (28.9)	.611
Personal history of breast cancer	2 (8.0)	18 (11.8)	.743
Met FPC^1^ criteria	4 (16.0)	26 (17.1)	.891
Met NCCN^2^ criteria for BRCA testing	11 (44.0)	57 (37.5)	.536
Met either FPC or NCCN criteria	11 (44.0)	71 (46.7)	.801
Stage of cancer			.341
Resectable	4 (16.0)	48 (31.6)	
Borderline resectable	2 (8.0)	14 (9.2)	
Locally advanced	9 (36.0)	35 (23.0)	
Metastatic	10 (40.0)	55 (36.2)	
Primary resection	5 (20.0)	55 (36.2)	.113
ECOG			.481
0	5 (20.0)	35 (23.0)	
1	13 (52.0)	78 (51.3)	
2	7 (28.0)	28 (18.4)	
3	0 (0.0)	11 (7.2)	
Family history			
PDAC	4 (16.0)	38 (25.0)	.327
Breast cancer	10 (40.0)	57 (37.5)	.811
Ovarian cancer	1 (4.0)	13 (8.6)	.696
Prostate cancer	5 (20.0)	29 (19.1)	1.000
Colon cancer	9 (36.0)	39 (25.7)	.281
Melanoma	4 (16.0)	8 (5.3)	.070

^1^ FPC = familial pancreatic cancer.

^2^ NCCN = National Comprehensive Cancer Network version 2017.

PVs were identified in 25/177 (14.1%) cases of the referred, unrelated PDAC cohort affecting ten different genes: *ATM* (6.8%, N = 12, including two *ATM* cases that each had a PV in a second cancer susceptibility gene (*ATM/BRCA2* and *ATM/SDHA*)); *BRCA2* (2.3%, N = 4, including the aforementioned case with a second PV in *ATM*); *CDKN2A* (1.1%, N = 2); *MSH2* (1.1%, N = 2); *CHEK2* (1.1%, N = 2); *SDHA* (0.6%, N = 1, the case with a second PV in *ATM*); *BRIP1* (0.6%, N = 1)*;* monoallelic *MUTYH* (0.6%, N = 1); *MITF* (0.6%, N = 1); and *NBN* (0.6%, N = 1) (Table S2). We found PV in known PDAC susceptibility genes in 19/177 patients (10.7%) overall with cascade screening implications that can alter management of healthy at‐risk relatives (*ATM* (10), *ATM*/*SDHA* (1), *BRCA2* (3), *BRCA2*/*ATM* (1), *CDKN2A* (2), and *MSH2* (2)). Large deletions were detected in two patients (1.1%) involving the *ATM* gene (deletion of exon 9) and the *MSH2* gene (deletion of exon 1). Variants of uncertain significance (VUS) were identified in 20 genes among 31 patients (17.5%, 31/177) (Table S3). Variant frequency from Color Genomics Data can be found at data.color.com/v1.

### 3.1 Case‐control analysis

Cases tested on the Color Genomics platform were matched to controls from Color Genomics Database. As shown in Table 3, there was a significant association between *ATM* and the risk of pancreatic cancer (OR, 7.73; 95% CI, 3.10 to 19.33, *P* = 6.14E‐05). The odds ratios for PV in other known PC susceptibility genes were not significant in this case‐control analysis, however, the study was under‐powered due to small sample size to be able to make conclusions on the associations.

**Table 3:** Comparisons between PDAC cases (N = 164) and control cases (N = 1342)

Gene	PDAC cases with PV N (%)	Color controls with PV N (%)	Odds Ratio (95% CI)	*P* value
*ATM*	9 (5.5)	10 (0.7)	7.73 (3.10‐19.33)	6.14E‐05
*NBN*	1 (0.6)	6 (0.4)	1.37 (0.16‐11.42)	.555
*MUTYH*	1 (0.6)	22 (1.6)	0.37 (0.05‐2.75)	.502
*CDKN2A*	1 (0.6)	2 (0.1)	4.11 (0.37‐45.58)	.293
*CHEK2*	2 (1.2)	23 (1.7)	0.71 (0.17‐3.03)	1.000
*BRIP1*	1 (0.6)	3 (0.2)	2.74 (0.28‐26.48)	.370
*BRCA2*	2 (1.2)	21 (1.6)	0.78 (0.18‐3.34)	1.000
*MITF*	1 (0.6)	5 (0.4)	1.64 (0.19‐14.13)	.500
*MSH2*	1 (0.6)	2 (0.1)	4.11 (0.37‐45.58)	.293

As shown in Table S2, two of the *ATM* variants classified to be of uncertain significance by Color Genomics have been classified as pathogenic or likely pathogenic by other clinical labs and thus were included in the overall PV rate. However, they were considered as VUS for the case‐control analysis to be consistent with the testing laboratory's classification.

### 3.2 Genetic testing implications

Utilizing FPC or NCCN criteria did not appear, at least in this referral‐based study, to select for patients with a higher risk of PV with only 11/80 (13.8%) PV identified vs 14/97 (14.4%) in those that did not meet either criteria (*P* = .897). These criteria would have missed 56% (14/25) of patients with PV in various genes (*ATM* (6), *ATM*/*SDHA* (1), *BRCA2*/*ATM* (1), *BRCA2* (1), *MSH2* (2), *NBN* (1), *MITF* (1), and *CHEK2* (1)). All 25 patients were the first affected family member to have germline PV identified in their respective families. The overall number of first‐degree and second‐degree relatives at risk for the PV is 95 and 148, respectively. To date, 8/25 of families have accessed carrier testing for a total of 35 relatives within the province; testing completed outside of province was not captured.

Six PDAC patients who completed testing during the study period were excluded from our index cohort analysis including five who were referred for carrier testing for a PV previously identified in another family member. They all tested positive for the familial PV. There was a median of 4.5 years from family index testing to carrier testing (range of 2 months to 19 years). The sixth case was referred for site‐specific mutation testing to confirm a *BRCA2* mutation detected through another research program that utilized whole‐genome sequencing. We compared the baseline characteristics between carrier cases and our index cohort (Table S4). Factors of age at diagnosis, FPC criteria, NCCN criteria for BRCA testing, and family history of PDAC and melanoma were significantly different between the two cohorts.

## 4 DISCUSSION

This study used a widely available, clinical‐grade 30‐gene NGS panel in a prospective clinic‐based PDAC cohort referred for genetic testing. Testing was offered to all referred patients unselected for family history and demonstrated an overall hereditary cancer PV detection rate of 14.1%. When excluding genes not clearly associated with pancreatic cancer, the mutation detection rate is 10.7%, which is similar to previous reports.^2^, ^4^, ^16^, ^17^, ^18^, ^19^ Although the significance of germline variants in *BRIP1*, *CHEK2*, and monoallelic *MUTYH* within the context of PDAC remains uncertain, the implications with regard to other hereditary cancer risks remain clinically meaningful and demonstrate the utility of wider gene panel testing. Furthermore, these findings could have potential future relevance as an increased understanding of their contribution to various cancers evolve.^20^ A recent study on 289 resected PDAC cases unselected for personal or family history characteristics with a 9.7% germline mutation rate using a 24 gene panel showed that fewer than half of the germline cases had an identified second hit in the wild‐type allele of the tumor by paired somatic analysis.^21^ Importantly, in our study, structural variants were seen in 2/25 (8%) of patients with PV which has important implications for labs conducting sequencing studies that may have difficulty identifying these types of variants.

An important limitation is the referral‐based nature of the study. We observed that PDAC patients in our study, when compared to the provincial PDAC population from BC Cancer statistics^22^ were more likely to be female and to be younger at diagnosis. Although patients in our study came from all five provincial health authorities, there was an unequal proportion from the health authority in which our program is located. However, this does represent a real‐world experience of referral patterns for genetic testing. A family history of pancreatic cancer, usually defined as having at least one affected first degree relative is seen in 5% to 10% of individuals with this disease.^23^ However, 46.2% of this study cohort met either NCCN or FPC testing criteria.

Our cohort demonstrated an enrichment of *ATM* PV (6.8%) in BC, Canada. Our findings derived from case‐control analysis of samples tested on the same platform, validate the association seen in *Hu et al* OR for *ATM* PV of 5.71 (95% CI, 4.38 to 7.33), in a Canadian population that is relatively diverse comprising 29.4% non‐Caucasian and 1.2% Ashkenazi Jewish ancestry.^16^ In light of this, adoption of extended multigene panel testing for PDAC should cover *ATM*. Our study had > 90% post‐hoc power to assess the association between *ATM* and PDAC. The number of PV in other genes was too low to allow for evaluation of associations with pancreatic cancer.

There was heterogeneity of germline tests used in this study. Although most patients did receive the 30 gene panel (92.6%), the 30 gene panel has limited coverage of the *PMS2* gene, specifically exons 12‐15, however, it is unlikely that this limitation would have significantly impacted the overall mutation detection rate.

Baseline demographics were similar between patients with and without PV though sample size represents a limitation of our study. As reported by others,^6^ traditional screening criteria do not perform well for identifying PV in PDAC and would have missed 56% patients with PVs in our study including those in *BRCA2*, *ATM*, and *MSH2*, which have implications for cancer risk‐reduction strategies and screening and surveillance in healthy family members and for which management guidelines have been developed_._ 24, 25 Based on these guidelines, healthy relatives carrying the familial *ATM* mutation, for example, who have a moderately to strongly increased risk for breast and a moderately increased risk for pancreatic cancer are eligible for consideration of publicly funded annual breast MRI screening starting at age 40 and annual pancreatic cancer screening with endoscopic ultrasound and alternating MRI of the pancreas beginning at age 50 (or 10 years before the youngest diagnosis of pancreatic cancer in the family). Using a simple age criterion of PDAC diagnosis ≤ 50 years would have missed 19/25 patients with PV. Median overall survival (mOS) was similar to published literature though the metastatic group seemed to have slightly superior survival with mOS 13 months (95% CI, 11 to 15).^26^, ^27^, ^28^ Testing in metastatic PDAC patients did not enrich for germline PV detection in our cohort. In summary, clinicopathological features cannot be relied upon as criteria for pancreatic genetic testing and our unselected PDAC testing strategy revealed a hereditary cancer syndrome in 25 families.

Given the significant morbidity and mortality of pancreatic cancer, implementation of universal testing for PDAC patients will require efficient genetic testing protocols. Within our referral based research program, only 59% of all referred patients completed genetic testing. This is similar to the 60% test uptake rate seen in a recent publication on 137 patients with PDAC referred to a clinical genetics service.^29^ In that study, common reasons for not completing testing were worsening disease severity, lack of patient follow‐up, insurance concerns, and logistic/travel challenges.^29^ In our study, despite scheduling consultations within 3 months of referral, 12.8% (N = 39) of patients died prior to their HCP appointment or prior to submitting a sample for genetic testing. Patients who did not meet criteria for publicly funded testing may have felt overburdened by the additional requirement for research consent forms. Telehealth appointments were offered to remove potential logistical barriers for patients who lived at a distance or felt too unwell to attend in person. However, our data show a significantly lower rate of testing uptake among the telehealth group, which will require further examination. Given the study limitation that appointment types were partly driven by patient choice, differences in uptake may not directly relate to the genetic counselling method received, but could reflect underlying associations as to why patients chose each method, such as rural vs urban geographic location. Although previous studies have shown a lower uptake of testing in telehealth compared to in‐person genetic counseling, there were no differences in psychosocial outcomes.^30^ In our study, the difference in test uptake did not appear to be explained by a difference in performance status or disease stage in the telehealth group and it is possible that logistics for international courier delivery was a barrier to testing for some patients. Point of care testing has demonstrated that the convenience of testing is a strong determinant of testing uptake in this population.^31^

Based on this study, if universal testing of PDAC were to be publicly funded in BC, it would mean a five‐fold increase in publicly funded tests for PDAC patients and accommodation of associated clinical follow‐up. We successfully implemented group information sessions as a service delivery model in this study population, which resulted in reduced wait times, less resources utilised, high satisfaction and uptake of testing. A group model could be scaled up going forward. However, given the overall suboptimal testing uptake in the genetics referral based protocol and in light of emerging treatment implications of *BRCA1*/*BRCA2* PV, mismatch repair deficiency and potentially other defects in DNA repair, another avenue to improve genetic testing rates in patients with PDAC may be oncologist‐directed genetic testing, similar to the mainstreaming approach undertaken in breast and ovarian cancer or upfront tumor or tumor normal sequencing approaches, which may have different considerations for consent.^32^, ^33^, ^34^ Local experience with the oncologist‐directed genetic testing model resulted in similar patient‐reported outcomes, was acceptable to health care providers, and significantly reduced wait times for genetic testing as compared to a traditional 1‐on‐1 approach.^35^

Among the 5 cases who presented for carrier testing only after the diagnosis of pancreatic cancer, four of the individuals would have been eligible for pancreatic screening according to the International Cancer of the Pancreas Screening Consortium guidelines^36^ and in 1 case, the patient was the first PDAC diagnosis in the family. Supporting oncologist‐initiated genetic testing with subsequent genetics referrals for PDAC patients found to carry PV or VUS, may not only improve rates of testing, but may also allow refocusing of clinical genetics resources towards genetic risk stratification in PV‐positive families, prevention and early pancreatic cancer detection in at‐risk relatives.

## 5 CONCLUSION

Within the prospective clinic‐based cohort of patients with PDAC, unselected for family history, germline PV were detected in 14.1% (25/177) of patients undergoing index genetic testing. Prior criteria‐based genetic testing would have missed a substantial proportion of PV, which can carry therapeutic implications for patients and screening implications for at‐risk healthy relatives. Universal genetic testing in PDAC using a multigene panel approach should be adopted with consideration of the logistical challenges to implementation that remain a significant barrier to best practice.

## CONFLICT OF INTEREST

CLN, WS, and AYZ are employed by and have equity interest in Color Genomics. DFS reports consulting fees from Robarts Clinical Trials Inc. KAS reports consulting fees from AstraZeneca. All other authors have no conflicts of interest to declare.

## AUTHOR CONTRIBUTIONS

Kasmintan A. Schrader designed and directed the study, and provided supervision. Daniel J. Renouf, Sophie Sun, and David F. Schaeffer provided supervision. Carol Cremin contributed to clinical specimen and data collections. Carol Cremin and Michael Kuan‐Ching Lee developed the original draft of the manuscript. Carol Cremin, Michael Kuan‐Ching Lee, Quan Hong, Cynthia L. Neben, Will Stedden, Alicia Y. Zhou, and Kasmintan A. Schrader performed data analysis, and interpretation. Carol Cremin, Michael Kuan‐Ching Lee, Quan Hong, Carolyn Hoeschen, Anna Mackenzie, Katherine Dixon, Mary McCullum, Jennifer Nuk, Steve Kalloger, Joanna Karasinska, Charles Scudamore, Peter T.W. Kim, Fergal Donnellan, Eric C.S. Lam, Howard J. Lim, David F. Schaeffer, Sophie Sun, Daniel J. Renouf, and Kasmintan A. Schrader participated in the writing of the article.

## Supporting information

Fig S1

Table S1

Table S2

Table S3

Table S4

Table S5

## Data Availability

The data that support the findings of this study are available on request from the corresponding author. The data are not publicly available due to privacy or ethical restrictions.
