# Supplementary material for: Burden of hereditary cancer susceptibility in unselected patients with pancreatic ductal adenocarcinoma referred for germline screening
Source: Cancer Med. 2020 Apr 7;9(11):4004–13. doi: 10.1002/cam4.2973 (PMC7286471; doi:10.1002/cam4.2973)
Supplement: Supplementary file 1 — Fig S1 [file CAM4-9-4004-s001.docx]

**Supplementary Figure 1.** Overall survival analysis. Survival of patients with PV detected (*N*=25) versus uninformative group (*N*=152). Patient survival based on time of diagnosis to time of death or date of censor on 31^st^ March 2019.
