# Supplementary material for: Burden of hereditary cancer susceptibility in unselected patients with pancreatic ductal adenocarcinoma referred for germline screening
Source: Cancer Med. 2020 Apr 7;9(11):4004–13. doi: 10.1002/cam4.2973 (PMC7286471; doi:10.1002/cam4.2973)
Supplement: Supplementary file 2 — Table S1 [file CAM4-9-4004-s002.docx]

**Supplementary Table 1.** Gene panels used for each index pancreatic ductal adenocarcinoma (PDAC) case (*N*=177)

| **Tested patients (*N*)** | **1** | **1** | **1** | **2** | **4** | **1** | **163** | **1** | **2** | **1** |
| --- | --- | --- | --- | --- | --- | --- | --- | --- | --- | --- |
| genes tested in each panel (*n*) | 95 | 83 | 68 | 61 | 57 | 42 | 30 | 26 | 17 | 1 |
| *AIP* |  |  | * |  |  |  |  |  |  |  |
| *ALK* | * | * | * |  |  |  |  |  |  |  |
| *APC* | * | * | * | * | * | * | * | * | * |  |
| *ATM* | * | * | * | * | * | * | * | * |  |  |
| *AXIN2* | * | * |  |  |  | * |  |  |  |  |
| *BAP1* | * | * | * | * | * |  | * | * |  |  |
| *BARD1* | * | * | * | * | * | * | * |  |  |  |
| *BLM* | * | * | * |  |  |  |  |  |  |  |
| *BMPR1A* | * | * | * | * | * | * | * | * | * |  |
| *BRCA1* | * | * | * | * | * | * | * | * | * |  |
| *BRCA2* | * | * | * | * | * | * | * | * | * | * |
| *BRIP1* | * | * | * | * | * | * | * |  |  |  |
| *CASR* | * | * |  |  |  |  |  |  |  |  |
| *CDC73* | * | * |  | * | * |  |  |  |  |  |
| *CDH1* | * | * | * | * | * | * | * |  | * |  |
| *CDK4* | * | * | * | * | * |  | * | * |  |  |
| *CDKN1B* | * | * | * |  |  |  |  |  |  |  |
| *CDKN1C* | * | * |  |  |  |  |  |  |  |  |
| *CDKN2A* | * | * | * | * | * | * | * | * |  |  |
| *CEBPA* | * | * |  |  |  |  |  |  |  |  |
| *CHEK2* | * | * | * | * | * | * | * |  |  |  |
| *CTNNA1* |  | * |  |  |  |  |  |  |  |  |
| *DICER1* | * | * | * | * | * | * |  |  |  |  |
| *DIS3L2* | * | * |  |  |  |  |  |  |  |  |
| *EGFR* | * | * |  |  |  |  |  |  |  |  |
| *EPCAM* | * | * | * | * | * | * | * | * |  |  |
| *FH* | * | * | * | * | * |  |  |  |  |  |
| *FLCN* | * | * | * | * | * |  |  |  |  |  |
| *GATA2* | * | * |  |  |  |  |  |  |  |  |
| *GPC3* | * | * |  |  |  |  |  |  |  |  |
| *GREM1* | * | * | * | * | * | * | * |  |  |  |
| *HOXB13* | * | * | * | * | * |  |  |  |  |  |
| *HRAS* | * | * |  |  |  |  |  |  |  |  |
| *KIT* | * | * |  | * | * | * |  |  |  |  |
| *MAX* | * | * | * | * | * |  |  |  |  |  |
| *MEN1* | * | * | * | * | * | * |  | * |  |  |
| *MET* | * | * | * | * | * |  |  |  |  |  |
| *MITF* | * | * | * | * | * |  | * | * |  |  |
| *MLH1* | * | * | * | * | * | * | * | * | * |  |
| *MSH2* | * | * | * | * | * | * | * | * | * |  |
| *MSH3* |  | * |  | * |  |  |  |  |  |  |
| *MSH6* | * | * | * | * | * | * | * | * | * |  |
| *MUTYH* | * | * | * | * | * | * | * |  | * |  |
| *NBN* | * | * | * | * | * | * | * |  |  |  |
| *NF1* | * | * | * | * |  | * |  | * |  |  |
| *NF2* | * | * | * | * | * |  |  |  |  |  |
| *NTHL1* |  | * | * | * |  |  |  |  |  |  |
| *PALB2* | * | * | * | * | * | * | * | * | * |  |
| *PDGFRA* | * | * |  | * | * | * |  |  |  |  |
| *PHOX2B* | * | * | * |  |  |  |  |  |  |  |
| *PMS2* | * | * | * | * | * | * | * | * | * |  |
| *POLD1* | * | * | * | * | * | * | * |  | * |  |
| *POLE* | * | * | * | * | * | * | * |  | * |  |
| *POT1* | * | * | * |  |  |  |  | * |  |  |
| *PRKAR1A* | * | * | * | * | * |  |  |  |  |  |
| *PTCH1* | * | * | * | * | * |  |  |  |  |  |
| *PTEN* | * | * | * | * | * | * | * | * | * |  |
| *RAD50* | * | * | * |  |  | * |  |  |  |  |
| *RAD51C* | * | * | * | * | * | * | * |  |  |  |
| *RAD51D* | * | * | * | * | * | * | * |  |  |  |
| *RB1* | * | * | * | * | * |  |  | * |  |  |
| *RECQL4* | * | * |  |  |  |  |  |  |  |  |
| *RET* | * | * | * | * | * |  |  |  |  |  |
| *RUNX1* | * | * |  |  |  |  |  |  |  |  |
| *SDHA* | * | * | * | * | * | * |  |  |  |  |
| *SDHAF2* | * | * | * | * | * |  |  |  |  |  |
| *SDHB* | * | * | * | * | * | * |  |  |  |  |
| *SDHC* | * | * | * | * | * | * |  |  |  |  |
| *SDHD* | * | * | * | * | * | * |  |  |  |  |
| *SMAD4* | * | * | * | * | * | * | * | * | * |  |
| *SMARCA4* | * | * | * | * | * | * |  |  |  |  |
| *SMARCB1* | * | * | * | * | * |  |  |  |  |  |
| *SMARCE1* | * | * | * |  |  |  |  |  |  |  |
| *STK11* | * | * | * | * | * | * | * | * | * |  |
| *SUFU* | * | * | * |  |  |  |  |  |  |  |
| *TERC* | * | * |  |  |  |  |  |  |  |  |
| *TERT* | * | * |  |  |  |  |  |  |  |  |
| *TMEM127* | * | * | * | * | * |  |  |  |  |  |
| *TP53* | * | * | * | * | * | * | * | * | * |  |
| *TSC1* | * | * | * | * | * | * |  | * |  |  |
| *TSC2* | * | * | * | * | * | * |  | * |  |  |
| *VHL* | * | * | * | * | * | * |  | * |  |  |
| *WRN* | * | * |  |  |  |  |  |  |  |  |
| *WT1* | * | * |  | * | * |  |  |  |  |  |
| *BUB1B* | * |  |  |  |  |  |  |  |  |  |
| *CEP57* | * |  |  |  |  |  |  |  |  |  |
| *ERCC4* | * |  |  |  |  |  |  |  |  |  |
| *FANCA* | * |  |  |  |  |  |  |  |  |  |
| *FANCB* | * |  |  |  |  |  |  |  |  |  |
| *FANCC* | * |  | * |  |  |  |  |  |  |  |
| *FANCD2* | * |  |  |  |  |  |  |  |  |  |
| *FANCE* | * |  |  |  |  |  |  |  |  |  |
| *FANCF* | * |  |  |  |  |  |  |  |  |  |
| *FANCG* | * |  |  |  |  |  |  |  |  |  |
| *FANCI* | * |  |  |  |  |  |  |  |  |  |
| *FANCL* | * |  |  |  |  |  |  |  |  |  |
| *FANCM* | * |  |  |  |  |  |  |  |  |  |
| *SLX4* | * |  |  |  |  |  |  |  |  |  |
| *XRCC2* | * |  | * |  |  |  |  |  |  |  |
| *GALNT12* |  |  | * |  |  |  |  |  |  |  |
| *MRE11A* |  |  | * |  |  |  |  |  |  |  |
| *AXIN1* |  |  |  | * |  |  |  |  |  |  |
