# Supplementary material for: Burden of hereditary cancer susceptibility in unselected patients with pancreatic ductal adenocarcinoma referred for germline screening
Source: Cancer Med. 2020 Apr 7;9(11):4004–13. doi: 10.1002/cam4.2973 (PMC7286471; doi:10.1002/cam4.2973)
Supplement: Supplementary file 3 — Table S2 [file CAM4-9-4004-s003.docx]

**Supplementary Table 2**. Germline pathogenic or likely pathogenic variants identified in the index PDAC cohort (*N*=177)

| **Gene** | **HGVS** | **Sex^a^** | **Ethnicity** | **FPC^b^** | **NCCN^c^** | **Frequency in Color Data (%)** |
| --- | --- | --- | --- | --- | --- | --- |
| *ATM* | c.387delA (p.Asp130Ilefs*23) | F | Asian | No | No | 0 |
| *ATM* | c.192dupA (p.Gln65Thrfs*35) | F | Asian | No | No | 0 |
| *ATM* | c.5644C>T (p.Arg1882*) | F | European | No | No | 0 |
| *ATM* | c.8565T>G (p.Ser2855Arg) | M | European | No | Yes | 0 |
| *ATM* | deletion of exon 9 | F | Asian | No | No | 0 |
| *ATM^1^* | c.5763-1050A>G | M | European | No | No | 0.01 |
| *ATM^2^* | c.967A>G (p.Ile323Val) | M | European | Yes | Yes | 0 |
| *ATM* | c.1783_1802+91del | M | European | No | No | 0 |
| *ATM* | c.7792C>T (p.Arg2598*) | M | Asian | No | Yes | 0.002 |
| *ATM* | c.4098_4099delTG (p.Cys1366*) | F | European | Yes | Yes | 0.004 |
| *ATM/SDHA* | ATM : c.5623C>T (p.Arg1875*) SDHA : c.223C>T (p.Arg75*) | F | . | No | No | 0, n/a |
| *BRCA2/ATM* | BRCA2 : c.6275_6276delTT (p.Leu2092Profs*7) ATM : c.944delT (p.Leu315Tyrfs*5) | F | European | No | No | 0.04, 0 |
| *BRCA2* | c.7133C>G (p.Ser2378*) | M | Asian | No | No | 0.008 |
| *BRCA2* | c.3545_3546delTT (p.Phe1182*) | F | European | No | Yes | 0.02 |
| *BRCA2* | c.8878C>T (p.Gln2960Ter) | F | European | No | Yes | 0.002 |
| *BRIP1* | c.1510dup (p.Ile504Asnfs*7) | F | European | No | Yes | 0 |
| *CDKN2A* | c.377T>A (p.Val126Asp) | M | European | Yes | Yes | 0 |
| *CDKN2A* | c.225_243del (p.Ala76Cysfs*64) | M | European | Yes | Yes | 0 |
| *CHEK2* | c.470T>C (p.Ile157Thr) | F | European | No | Yes | 0.70 |
| *CHEK2* | c.1100delC (p.Thr367Metfs*15) | M | European | No | No | 0.66 |
| *MITF* | c.952G>A (p.Glu318Lys) | M | European | Yes | No | 0.50 |
| *MSH2* | c.1786_1788delAAT (p.Asn596del) | M | European | No | No | 0 |
| *MSH2* | deletion exon 01, c.1-412-?_211+230+?del | F | European | No | No | 0 |
| *MUTYH* | c.934-2A>G | F | Asian | Yes | Yes | 0.13 |
| *NBN* | c.657_661delACAAA (p.Lys219Asnfs*16) | M | European | No | No | 0.10 |

1. Color Genomics classified this variant as VUS but other labs have considered it pathogenic (including Invitae and Ambry Genetics) or a “reduced penetrance” pathogenic allele (BC provincial lab). Patient was diagnosed with PDAC at 83; family history includes a daughter with breast cancer at 61 and daughter with melanoma in her 50s, no reported relatives with pancreatic cancer. This case was included in the case control analysis, using the original Color Genomics variant classification.

2. Color Genomics classified this variant as VUS but other labs have considered it likely pathogenic (including GeneDx and Counsyl). Patient was diagnosed with PDAC at 65; family history includes a father with prostate cancer at 63, two paternal cousins with pancreatic cancer and a paternal cousin with bilateral breast cancer at 43 and 57, the latter patient was also confirmed to carry the ATM variant. This case was included in the case control analysis, using the original Color Genomics variant classification.

^a^ F=female, M=male.

^b^ FPC = familial pancreatic cancer.

^c^ NCCN = National Comprehensive Cancer Network, version 2017.
