# Supplementary material for: Burden of hereditary cancer susceptibility in unselected patients with pancreatic ductal adenocarcinoma referred for germline screening
Source: Cancer Med. 2020 Apr 7;9(11):4004–13. doi: 10.1002/cam4.2973 (PMC7286471; doi:10.1002/cam4.2973)
Supplement: Supplementary file 4 — Table S3 [file CAM4-9-4004-s004.docx]

**Supplementary Table 3. Germline variants of uncertain significance identified in the index PDAC cohort (*N*=177)**

| **Gene** | **HGVS** | **Sex^a^** | **Ethnicity** | **FPC^b^** | **NCCN^c^** | **Frequency in Color Data (%)** |
| --- | --- | --- | --- | --- | --- | --- |
| *APC* | c.4072G>A (p.Ala1358Thr) | F | European | No | Yes | 0.02 |
| *APC* | c.7918T>C (p.Ser2640Pro) | F | European | No | No | 0 |
| *APC* | c.3377G>A (p.Ser1126Asn) | F | European | No | No | 0 |
| *APC/BRCA2/NBN* | APC c.5404G>A (p.Glu1802Lys) BRCA2 c.7100C>T (p.Thr2367Ile) NBN c.1160C>G (p.Ser387Cys) | F | Asian | No | Yes | 0, 0.002, 0 |
| *ATM* | c.1379C>T (p.Thr460Met) | F | European | No | No | 0.002 |
| *ATM* | c.5753G>C (p.Arg1918Thr) | M | European | No | No | 0.02 |
| *ATM/MSH2/RAD50* | ATM c.1511A>G (p.Asn504Ser) MSH2 c.1369A>G (p.Thr457Ala) RAD50 c.3143T>C (p.Met1048Thr) | F | Asian | No | Yes | 0, 0, n/a |
| *BAP1/BRIP1/PTCH1/PTEN* | BAP1 c.1045A>G (p.N349D) BRIP1 c.1018C>T (p.L340F) PTCH1 c.3955C>T (p.R1319C) PTEN c.-928C>T | M | Other | No | Yes | 0.0002, 0, n/a, 0 |
| *BAP1/PALB2/RAD51D* | BAP1 c.2158C>T (p.Arg720Cys) PALB2 c.2289G>C (p.Leu763Phe) RAD51D c.619T>C (p.Ser207Pro) | F | Asian | No | No | 0, 0.05, 0.0002 |
| *BARD1/CHEK2* | BARD1 c.91C>T (p.Arg31Cys) CHEK2 c.170C>T (p.Ser57Phe) | M | European | No | No | 0, 0 |
| *BRCA1* | c.1096G>C (p.Asp366His) | M | European | No | No | 0.004 |
| *BRCA1/MSH6* | BRCA1 c.1768A>T (p.Ser590Cys) MSH6 c.3740C>G (p.Thr1247Ser) | M | European | Yes | Yes | 0, 0 |
| *BRCA2* | c.9604C>T (p.Pro3202Ser) | M | European | Yes | Yes | 0.002 |
| *BRIP1* | c.3431A>G (p.Glu1144Gly) | M | Other | No | No | 0.004 |
| *BRIP1* | c.2830C>G (p.Gln944Glu) | M | Asian | No | Yes | 0.04 |
| *BRIP1* | c.2325T>G (p.Asn775Lys) | F | European | Yes | Yes | 0.002 |
| *CHEK2* | c.73G>A (p.Val25Ile) | M | Asian | No | No | 0.002 |
| *MLH1* | c.704A>T (p.Asp235Val) | F | Asian | Yes | Yes | 0 |
| *MSH2* | c.286C>T (p.Arg96Cys) | F | European | No | No | 0 |
| *MSH2* | c.14C>A (p.Pro5Gln) | M | Asian | No | No | 0.008 |
| *MUTYH* | c.667A>G (p.Ile223Val) | F | European | No | No | 0.07 |
| *MUTYH* | c.1588G>T (p.Asp530Tyr) | F | European | No | No | 0.03 |
| *PALB2* | c.1058A>G (p.Lys353Arg) | F | European | No | Yes | 0 |
| *PALB2* | c.149A>C (p.Lys50Thr) | M | Asian | No | No | 0.002 |
| *PALB2* | c.1250C>A (p.Ser417Tyr) | F | European | No | No | 0.04 |
| *PMS2* | c.779_780delCCinsTG (p.Ser260Leu) | M | Asian | No | No | 0 |
| *PMS2* | c.1249A>C (p.Ile417Leu) | M | European | No | Yes | 0 |
| *PMS2* | c.101G>T (p.Ser34Ile) | M | European | No | No | 0.01 |
| *POLD1* | c.311A>T (p.Tyr104Phe) | M | European | Yes | Yes | n/a |
| *PTEN* | c.1061C>A (p.Pro354Gln) | F | European | No | No | 0.02 |
| *RAD51D* | c.932T>A (p.Ile311Asn) | F | Asian | Yes | No | 0.05 |

^a^ F=female, M=male.

^b^ FPC = familial pancreatic cancer.

^c^ NCCN = National Comprehensive Cancer Network, version 2017.
