# Supplementary material for: Burden of hereditary cancer susceptibility in unselected patients with pancreatic ductal adenocarcinoma referred for germline screening
Source: Cancer Med. 2020 Apr 7;9(11):4004–13. doi: 10.1002/cam4.2973 (PMC7286471; doi:10.1002/cam4.2973)
Supplement: Supplementary file 5 — Table S4 [file CAM4-9-4004-s005.docx]

**Supplementary Table 4**. Comparisons between index (*N*=177) and carrier (*N*=5) patients

| **Characteristics** | **Carrier**  ***N* (%)** | **Index**  ***N* (%)** | ***P* value** |
| --- | --- | --- | --- |
| Age at diagnosis, years (Median, range) | 57 (36- 63) | 64 (36-89) | **0.049** |
| Gender  Female  Male | 4 (80.0)  1 (20.0) | 98 (55.4)  79 (44.6) | 0.387 |
| Ethnicity  European  Asian  Ashkenzai Jewish  Other  (Missing data) | 3 (60.0)  0 (0.0)  0 (0.0)  2 (40.0)  - | 124 (70.1)  38 (21.5)  3 (1.7)  7 (4.0)  5 (2.8) | 0.056 |
| Diabetes  Long term (>3yrs)  Peripancreatic (<3yrs)  No | 1 (20.0)  0 (0.0)  4 (80.0) | 24 (13.6)  17 (9.6)  136 (76.8) | 0.731 |
| Prior smoking history | 3 (60.0) | 74 (41.8) | 0.652 |
| Personal history of other cancer | 0 (0.0) | 50 (28.2) | 0.325 |
| Personal history of breast cancer | 0 (0.0) | 20 (11.3) | 1.000 |
| Met FPC^1^ criteria | 4 (80.0) | 30 (16.9) | **0.005** |
| Met NCCN^2^ criteria for BRCA testing | 5 (100.0) | 68 (38.4) | **0.010** |
| Met either FPC or NCCN criteria | 5 (100.0) | 82 (46.3) | **0.023** |
| Stage of cancer  Resectable  Borderline resectable  Locally advanced  Metastatic | 2 (40.0)  1 (20.0)  0 (0.0)  2 (40.0) | 52 (29.4)  16 (9.0)  44 (24.9)  65 (36.7) | 0.458 |
| Primary resection | 2 (40.0) | 60 (33.9) | 1.000 |
| ECOG  0  1  2  3 | 1 (20.0)  4 (80.0)  0 (0.0)  0 (0.0) | 40 (22.6)  91 (51.4)  35 (19.8)  11 (6.2) | 0.882 |
| Family history  PDAC  Breast cancer  Ovarian cancer  Prostate cancer  Colon cancer  Melanoma | 4 (80.0)  2 (40.0)  2 (40.0)  0 (0.0)  2 (40.0)  3 (60.0) | 42 (23.7)  67 (37.9)  14 (7.9)  34 (19.2)  48 (27.1)  12 (6.8) | **0.015**  1.000  0.062  0.586  0.616  **0.004** |

^1^FPC = familial pancreatic cancer.

^2^NCCN = National Comprehensive Cancer Network version 2017.
