# Supplementary material for: Burden of hereditary cancer susceptibility in unselected patients with pancreatic ductal adenocarcinoma referred for germline screening
Source: Cancer Med. 2020 Apr 7;9(11):4004–13. doi: 10.1002/cam4.2973 (PMC7286471; doi:10.1002/cam4.2973)
Supplement: Supplementary file 6 — Table S5 [file CAM4-9-4004-s006.doc]

**Supplementary Table 5. Genetic Counselling Appointment Satisfaction Survey**

Please indicate whether you agree or disagree with the following statements**:**

|  | strongly  agree | agree | disagree | strongly disagree | doesn’t  apply |
| --- | --- | --- | --- | --- | --- |
| Overall, this appointment was helpful to me |  |  |  |  |  |
| My expectations were met |  |  |  |  |  |
| I understood the information that was presented |  |  |  |  |  |
| The counsellor made me feel at ease |  |  |  |  |  |
| I was able to ask questions |  |  |  |  |  |
| I understand what will happen after this session |  |  |  |  |  |
| I found this appointment upsetting |  |  |  |  |  |
| I would feel comfortable calling the genetic counsellor to follow up if needed |  |  |  |  |  |
| Potentially sensitive or uncomfortable issues were handled well |  |  |  |  |  |
| My questions were answered |  |  |  |  |  |
| I found this appointment stressful |  |  |  |  |  |
| I understand hereditary cancer better than I did before this appointment |  |  |  |  |  |
| The counsellor listened to what I had to say |  |  |  |  |  |
| Enough time was provided for the appointment |  |  |  |  |  |

Do you have any suggestions about how we could improve? Or any other comments?

Thank you for taking the time to complete this form. Your input is important.
